# Mapping Long-Term Functional Changes in Cerebral Blood Flow by Arterial Spin Labeling

**DOI:** 10.1371/journal.pone.0164112

**Published:** 2016-10-05

**Authors:** Tracy Ssali, Udunna C. Anazodo, Yves Bureau, Bradley J. MacIntosh, Matthias Günther, Keith St. Lawrence

**Affiliations:** 1 Lawson Health Research Institute, London, ON, Canada; 2 Department of Medical Biophysics, Western University, London, ON, Canada; 3 Sunnybrook Health Sciences Centre, Toronto, ON, Canada; 4 Fraunhofer Institute for Medical Image Computing MEVIS, Bremen, Germany; 5 Mediri GmbH, Heidelberg, Germany; Banner Alzheimer's Institute, UNITED STATES

## Abstract

Although arterial spin labeling (ASL) is appealing for mapping long-term changes in functional activity, inter-sessional variations in basal blood flow, arterial transit times (ATTs), and alignment errors, can result in significant false activation when comparing images from separate sessions. By taking steps to reduce these sources of noise, this study assessed the ability of ASL to detect functional CBF changes between sessions. ASL data were collected in three sessions to image ATT, resting CBF and CBF changes associated with motor activation (7 participants). Activation maps were generated using rest and task images acquired in the same session and from sessions separated by up to a month. Good agreement was found when comparing between-session activation maps to within-session activation maps with only a 16% decrease in precision (within-session: 90 ± 7%) and a 13% decrease in the Dice similarity (within-session: 0.75 ± 0.07) coefficient after a month. In addition, voxel-wise reproducibility (within-session: 4.7 ± 4.5%) and reliability (within-session: 0.89 ± 0.20) of resting grey-matter CBF decreased by less than 18% for the between-session analysis relative to within-session values. ATT variability between sessions (5.0 ± 2.7%) was roughly half the between-subject variability, indicating that its effects on longitudinal CBF were minimal. These results demonstrate that conducting voxel-wise analysis on CBF images acquired on different days is feasible with only modest loss in precision, highlighting the potential of ASL for longitudinal studies.

## Introduction

To study the efficacy of therapies in conditions with variable patient outcomes such as chronic pain[1], longitudinal studies, in comparison to cross-sectional designs, provide the added benefit of accounting for individual variability since each participant serves as their own control[2, 3]. Due to the coupling of neuronal activity and regional cerebral blood flow (CBF), one approach for monitoring longitudinal functional changes is to image CBF. The MRI-based technique, arterial spin labeling (ASL), is well-suited for this purpose because it is non-invasive, quantitative and, in principle, statistical mapping approaches can be applied to data sets from separate sessions to detect longitudinal changes in CBF at the voxel-wise level[4, 5]. This, however, can be challenging due in part to the limited spatial resolution of ASL, resulting in partial volume errors if there are inconsistencies in head position between sessions. In addition, the ASL signal can be affected by day-to-day fluctuations in basal blood flow and arterial transit times (ATTs). Each of these factors increases the likelihood of Type-I errors when comparing ASL images from different sessions, ultimately leading to uncertainties in the interpretation of CBF changes between sessions.

Previous studies have demonstrated good reproducibility and reliability of resting ASL data within time frames ranging from hours[2, 6], weeks[7] and months[8]. More recently, studies have shown good reproducibility across centres[9] and vendors[10]. While these studies indicate the applicability of ASL to longitudinal monitoring, all of them focused on region-of-interest (ROI) analysis. Voxel-wise analysis, on the other hand, bears greater clinical relevance as affected regions may not be known a priori. Studies have reported relatively uniform between-session standard deviation maps across grey matter[11, 12]; however, only one study by Borogovac et al. investigated the ability of ASL to detect activation-induced CBF changes over extended periods. In this case, CBF changes associated with a visually cued motor task were generated using rest and task images separated by a month. Although activation was found in expected regions associated with motor and visual stimuli, significant CBF changes were also apparent in voxels unrelated to the task[5].

The overall aim of this study was to show that by minimizing sources of variance between sessions, ASL has the sensitivity to detect voxel-wise changes in CBF over extended periods on an individual basis. The ability of ASL to detect regional CBF changes was assessed using a motor task (finger tapping) that produces well-defined activation in sensorimotor regions[13]. This provided a means of distinguishing between task-related activation and possible false positive activation generated from rest and task images collected on separate days. ASL images were collected in three sessions with the second and third sessions one week and one month following the first. Resting CBF data from the three sessions were used to calculate voxel-wise within- and between-session reproducibility and reliability. In addition, ATT images were acquired in each session to measure the between-session and between-subject variability.

## Materials and Methods

This study was approved by the Health Sciences Research Ethics Board of the University of Western Ontario and all volunteers provided written informed consent in compliance with the Tri-Council Policy Statement of Ethical Conduct for Research Involving Humans.

### Study Design

This study was conducted using young (under the age of 24), healthy right-handed volunteers. Prior to each session, participants were instructed to abstain from consuming coffee and food for at least 6 hours and alcohol for 24 hours due to their potential vasomotor effects[14]. The majority of scans were scheduled in the morning to minimize the effect of diurnal CBF fluctuations [2]. Acquisition of ASL data was conducted in separate rest and task periods, rather than using an alternating block design, in order to generate within- and between-session activation maps by concatenating data from different sessions. The task consisted of self-paced sequential finger tapping (right-hand), and participants performed the task twice in each session (i.e. two runs) with a five and a half min rest period preceding each task period.

To replicate the head position in each session, an immobilizing foam head mold (Smithers Medical Products, Alpha Cradle) was created for each participant on the first visit and reused on return visits. As a secondary step to further minimize registration errors, manual alignment was performed on the scanner based on the comparison of structural MRIs acquired in the first and subsequent sessions.

### Image Acquisition

Participants underwent three identical scanning sessions scheduled a week and a month from the first. Imaging was conducted using a Siemens 3T Biograph mMR scanner equipped with a 32-channel head coil (Siemens Medical Systems, Erlangen, Germany). High-resolution sagittal T1-weighted magnetization prepared rapid gradient echo (MPRAGE) images were acquired (repetition time (TR)/echo time (TE): 2000/2.98 ms, flip angle: 9°, field of view (FOV): 256 x 256 mm^2^, 176 slices, voxel size: 1 mm^3^ isotropic resolution, bandwidth: 238Hz/Px, and scan duration: 3:35 min). This imaging volume was used for manual on-line image alignment and spatial normalization of the ASL images to MNI co-ordinates. Axial turbo spin echo (TSE) T2-weighted images (TR/TE: 6100/10 ms, flip angle: 120°, FOV: 220 x 220 mm^2^, 31 slices, voxel size: 0.57 x 0.57 x 4 mm^3^, gap: 0.8 mm, bandwidth: 223Hz/Px, and scan duration: 2:14 min) were also acquired for manual on-line image alignment.

ASL images were acquired using a single-shot 3D gradient/spin-echo (GRASE) sequence with background suppression (TR/TE: 3500/22.76 ms, label duration: 1500 ms, post labeling delay (PLD): 1200 ms, FOV: 240 x 240 mm^2^, 24 axial slices, voxel size: 3.8 x 3.8 x 6 mm^3^, bandwidth: 2004Hz/Px, and scan duration: 11:12 min)[15]. Pseudo continuous labeling was applied 90 mm below the centre slice. For each 11:12 min run (~5 min rest and ~5 min task), a total of 96 control-tag pairs were acquired. Between runs, equilibrium magnetization (M0) images were acquired with the same GRASE sequence with no arterial labeling or background suppression and the TR set to 5000 ms (scan duration: 30 s). For ATT mapping, GRASE images with background suppression were acquired at five PLDs: 700, 1300, 1900, 2500 and 3100 ms, with 5 control-tag pairs per PLD (TR/TE: 6000/18.76 ms, FOV: 500 x 500 mm^2^, voxel size: 12 x 8 x 6 mm^3^, bandwidth: 2004Hz/Px and scan duration: 5 min). For ASL and ATT mapping sequences, the timings of the two non-selective inversion pulses used for background suppression were empirically determined based on the PLD [15, 16]. Background suppression times are given in the supporting information (Table A in S1 File). The total acquisition time was approximately 34 minutes.

### Image Processing

#### ASL Perfusion-Weighted Images

Images were checked for gross head motion: translations greater than 3 mm and rotations greater than 3° as defined by Wang et al.[17]. Using SPM8 (Wellcome Trust Centre for Neuroimaging, University College London, UK), raw pCASL and M0 data from all sessions were realigned to the first volume of the first session using a least squares approach and a six-parameter rigid body spatial transformation. Next, the time series from each session was aligned to the first volume of its respective session. These steps corrected for differences in head positioning between sessions as well as motion within a session. T1-weighted images were skull stripped using FSL BET[18](FMRIB Software Library, Functional Magnetic Resonance Imaging of the Brain Centre, University of Oxford, Oxford, UK) and segmented using the unified segmentation method[19]. Pair-wise subtraction was used to generate perfusion-weighted images (ΔM) that were co-registered to their respective skull-stripped T1-weighted images using a rigid body transformation. These images were smoothed with an isotropic Gaussian kernel (6 mm full width at half maximum).

Using MATLAB (2012a, The MathWorks, Natick, MA), ASL images were converted into units of blood flow (ml/100g/min) using a single compartment flow model[20]:

$$f=\frac{\Delta M\lambda e^{\frac{PLD}{T_{1a}}}}{2\alpha M_{0}T_{1a}(1-e^{-\frac{\left(\tau +PLD\right)}{T_{1a}}})}$$ (1)

where λ = blood/tissue water partition coefficient (0.9 g/ml)[21], α = labeling efficiency (86%)[22], τ = label duration (1500 ms), and T1a = longitudinal relaxation time of arterial blood (1650 ms)[23]. Deformation parameters generated in the segmentation step were used to transform CBF maps into MNI space.

#### ATT Images

The multiple-PLD data acquired to map ATT were realigned and motion corrected as described previously. Using ASLtbx, control and label images were pair-wise subtracted and a voxel-wise parametric fit of a one-compartment kinetic model was performed using the FSL FABBER estimation routine[24]. The model included spatial priors and 200 iterations. The ATT images were co-registered to their respective T1-weighted image volume using a rigid-body transformation, smoothed with a 6 mm FWHM Gaussian filter and normalized to the MNI template in SPM.

#### Assessment of Image Alignment

Transformations (translation and rotation) required to align ΔM images were determined using the parameters from the realignment step. Within-session motion was characterized in terms of the rigid transformations necessary to align the first image volume of the first run to the first image volume of the second run. Similarly, between-session motion was defined as the average transformation required to align the first image volume from the first session to the first image volume of the second and third sessions.

#### Reproducibility and Reliability of Resting Measures

Reproducibility was characterized using the within-subject coefficient of variation (wsCV)[25]:

$$wsCV(\%)=\frac{SD_{∆CBF}}{Mean_{CBF}}∙100$$ (2)

where *SD*_Δ*CBF*_ represents the standard deviation between repeated measurements and *Mean*_*CBF*_ is the average CBF across sessions. Reliability was measured using a two way mixed model intraclass correlation coefficient (ICC)[26]:

$$ICC=\frac{\sigma_{bs}^{2}}{\sigma_{bs}^{2}+\sigma_{se}^{2}+\sigma_{er}^{2}}$$ (3)

where $\sigma_{bs}^{2}$ is the between-subject variance, $\sigma_{se}^{2}$ is the systemic error (variance between the repeated measures), and $\sigma_{er}^{2}$ is the error variance (ICC range: 0 to 1, values > 0.75 are classified as excellent reliability)[27].

In order to calculate voxel-wise wsCV and ICC of resting CBF, estimates of within- and between-session variances were calculated for each voxel from a repeated measures ANOVA performed using MATLAB. A similar procedure was also applied to the ATT images to calculate the between-session and between-subject reproducibility.

In addition to the voxel-wise analysis, reliability and reproducibility was also assessed within ROIs based on tissue type (grey and white matter), major lobes (frontal, parietal, temporal, occipital lobe) and selected cortical and subcortical regions (anterior cingulate cortex, amygdala, hippocampus, insular cortex, posterior cingulate cortex, somatosensory cortex and thalamus). These ROIs were defined using the Automated Anatomical Labeling (AAL) atlas[28] within the WFU Pickatlas[29] toolbox in SPM8. Grey and white matter masks were generated by thresholding the corresponding SPM8 probability maps by 80% and 60%, respectively. In contrast to conventional ROI analysis where reliability and reproducibility is calculated using region averaged CBF values[30, 31], ROI estimates were generated by multiplying the corresponding ICC and wsCV images by dichotomous masks and averaging the values within the region. To assess the effect of day-to-day variability in global CBF, all noise analyses were performed on absolute CBF (aCBF) images and on CBF images normalized by the mean grey matter value (relative CBF or rCBF).

### Motor Activation

#### Activation Contrasts

Contrasts for motor activation were generated by concatenating task data with rest data from: (a) same session and run (within-session), (b) same session but different run (within-session_DR_), and (c) different sessions separated by 1 week, 3 weeks, or 1 month (between-sessions). A diagram of the study design is shown in the supporting information (Fig A in S1 File). Analyses were performed using aCBF and rCBF data sets. Since normalizing has no effect on within-session activation, only aCBF within-session activation was generated. Activation maps were generated using the standard first level GLM analysis in SPM8. Areas of activation were identified with the *t*-statistic after correction for multiple comparisons using FWE rate (*p* < .05) and no cluster size threshold.

#### Precision of Motor Task Activation

Precision was defined as the ratio of the number of correctly predicted positive cases (true positives, TP) to the sum of TP and incorrectly predicted positive cases (false positives, FP):

$$Precision=\frac{TP}{TP+FP}∙100$$ (4)

True positives were classified as within-session voxels in task-related motor regions (i.e. supplementary motor area, primary motor cortex and cerebellum) that were identified as having significant CBF increases by the GLM. Within-session activation represents the “best case” scenario because it is unaffected by repositioning or basal fluctuations. However, given the statistical approach used to define activation, the within-session activation does not represent true activation. Consequently, using the within-session activation map as the ground truth would introduce a bias when analyzing the between-session activation. To avoid this error, the binary TP mask was dilated using a 3x3x3 structuring element. That is, for a given background voxel (i.e. zero), if the structuring element and the TP mask overlap by at least one non-zero voxel, the background voxel is set to a value of one. This dilation represents a conservative increase in mask volume to account for variations in the activation pattern. All activated voxels outside the mask were considered to be FP. Precision was calculated for within-session_DR_ and between-session activation maps.

#### Overlap of Activation Maps

The relative overlap between activation maps was quantified by the Dice similarity coefficient[32]:

$$Dice=\frac{2*V_{overlap}}{V_{1}+V_{2}}$$ (5)

where V_1_ and V_2_ are the number of activated voxels in the ROIs being compared, and V_overlap_ is the number of activated voxels common to V_1_ and V_2_. Dice coefficients range from 0 to 1 where a value of 1 indicates complete agreement between the activated and non-activated regions for the two maps. The fidelity of between-session activation was assessed by comparison to Dice coefficients generated using the within-session activation maps.

#### Statistical Analysis

A two way repeated measures ANOVA was used to compare findings among the imaging sessions (Version 20.0, SPSS Inc., Armonk, NY). This was performed for the alignment parameters, precision, Dice coefficient and whole brain grey matter CBF and ATT values. Using SPM, a voxel-wise repeated measures ANOVA was performed on resting CBF and ATT images across sessions. Where appropriate, pair-wise comparisons were performed using the Bonferroni Correction. For all analyses, *p*-values less than .05 were considered significant.

## Results

Data were acquired from seven participants (five females, mean age: 22.6 ± 1.3 years). Mean separation was 7.1 ± 0.7 days between the first and second sessions, 23.0 ± 3.3 days between the second and third sessions, and 30.1 ± 3.5 days between the first and third sessions. The first participant did not perform the motor task properly in the first run of the first session, so this data set was removed. Five of the 21 scans took place in the afternoon.

### Alignment of Images

Average within-session transformations were 0.30 ± 0.50, 1.48 ± 0.37, 0.46 ± 0.66 mm in the x, y, and z directions, respectively, and 0.22 ± 0.08, 0.14 ± 0.10, 0.09 ± 0.08° for pitch, roll, and yaw rotations, respectively. Between-session translations and rotations were 0.45 ± 0.76 mm (x), 0.97 ± 0.19 mm (y), 0.67 ± 0.71 mm (z), 0.36 ± 0.38° (pitch), 0.23 ± 0.26° (roll), and 0.14 ± 0.28° (yaw). There were no significant differences between rotations or translations. In addition, the within-session transformation parameters were not significantly different from the between-sessions values.

### Analysis of Resting Cerebral Blood Flow

#### Mean Resting Blood Flow

**Fig 1:**
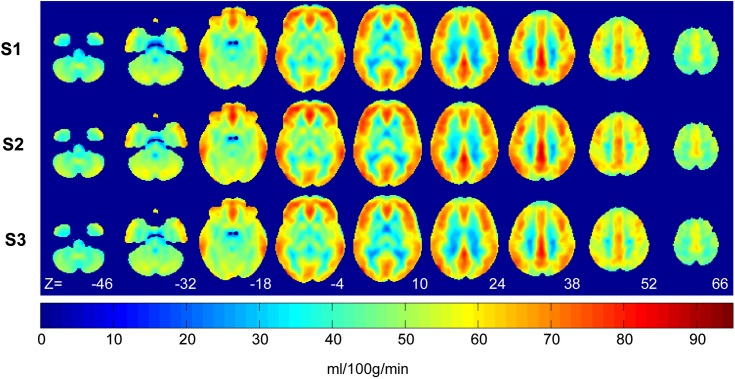
MNI-normalized images of group-averaged (N = 7) whole brain resting cerebral blood flow (ml/100g/min). Each session displayed, session 1(S1), session 2 (S2), and session 3 (S3), was the average of runs 1 and 2. Axial slice numbers (Z) in MNI co-ordinates are displayed at the bottom.

Fig 1 shows group-averaged whole brain resting aCBF maps for each session. Mean grey matter CBF was 55.9 ± 9.1, 58.2 ± 4.9, 56.0 ± 5.8 ml/100g/min for sessions 1, 2 and 3, respectively. There were no significant differences across sessions and, similarly, no significant voxel-wise CBF changes were detected. Since there was no significant main effect of CBF on session or run, comparisons between individual sessions were not assessed.

#### Reproducibility and Reliability of Resting Cerebral Blood Flow

**Fig 2:**
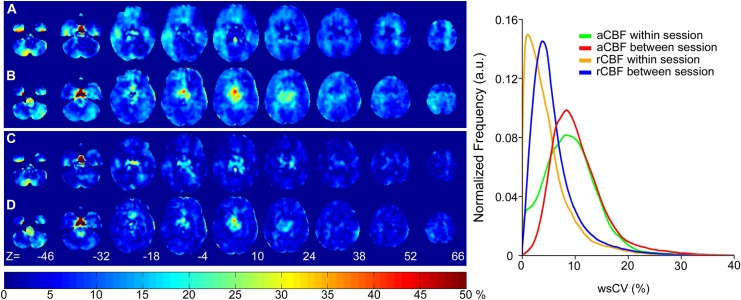
Voxel-wise whole brain average within-subject coefficient of variance (wsCV) maps calculated for absolute CBF (aCBF) and CBF normalized by grey matter CBF (rCBF). (A) within-session aCBF, (B) between-session aCBF, (C) within-session rCBF, and (D) between-session rCBF. Histograms were generated from each wsCV map.

Whole brain within-subject CV maps for within- and between-session resting CBF are shown in Fig 2. Within- and between-session reproducibility were similar, both having low wsCV values in cortical grey matter. From the aCBF images, mean voxel-wise wsCV across grey matter was 9.1 ± 5.2% for the within-session analysis and 10.0 ± 4.9% for between-session analysis. Normalizing the images by average grey matter CBF reduced the within-session wsCV to 4.7 ± 4.5% and the between-session wsCV to 5.7 ± 4.4%. In comparison to the rCBF wsCV histograms, aCBF wsCV histograms were broader and less left-skewed for both within-session and between-session analyses. The greater variability in aCBF is reflected by the intensity increase in Fig 2A and 2B relative to Fig 2C and 2D, which was also observed in the ROI analysis (Table 1A).

**Table 1:** Mean (± standard deviation) voxel-wise within and between-session: (A) wsCV and (B) ICC value for aCBF and rCBF images in select ROIs.

**A)** Within Subject Coefficient of Variation				
	**aCBF wsCV (%)**		**rCBF wsCV (%)**	
**ROI**	**Within**	**Between**	**Within**	**Between**
	**Session**	**Sessions**	**Session**	**Sessions**
Grey Matter	9.1 ± 5.2	10.0 ± 4.9	4.7 ± 4.5	5.7 ± 4.4
White Matter	9.8 ± 4.4	10.9 ± 4.4	4.4 ± 3.8	5.6 ± 3.4
Anterior Cingulate	11.2 ± 2.8	9.8 ± 3.5	3.1 ± 1.8	4.4 ± 2.3
Amygdala	8.6 ± 3.4	15.9 ± 4.3	4.2 ± 5.0	7.8 ± 3.3
Hippocampus	8.0 ± 4.8	12.3 ± 4.5	6.9 ± 6.5	6.5 ± 4.1
Insular Cortex	8.7 ± 4.1	7.7 ± 2.8	3.5 ± 2.4	5.0 ± 2.4
Posterior Cingulate	8.3 ± 5.0	13.6 ± 4.5	5.8 ± 5.7	6.2 ± 4.1
Somatosensory Cortex	9.2 ± 4.1	10.2 ± 4.2	3.5 ± 2.8	5.4 ± 3.5
Thalamus	5.1 ± 3.6	21.7 ± 8.1	13.4 ± 5.5	14.6 ± 7.3
**B)** Intraclass Correlation Coefficient				
	**aCBF ICC**		**rCBF ICC**	
**ROI**	**Within**	**Between**	**Within**	**Between**
	**Session**	**Sessions**	**Session**	**Sessions**
Grey Matter	0.85 ± 0.23	0.66 ± 0.19	0.89 ± 0.20	0.84 ± 0.15
White Matter	0.87 ± 0.17	0.69 ± 0.17	0.92 ± 0.13	0.86 ± 0.12
Anterior Cingulate	0.90 ± 0.06	0.66 ± 0.14	0.95 ± 0.04	0.84 ± 0.11
Amygdala	0.70 ± 0.27	0.34 ± 0.17	0.72 ± 0.28	0.64 ± 0.19
Hippocampus	0.68 ± 0.28	0.45 ± 0.18	0.68 ± 0.32	0.73 ± 0.2
Insular Cortex	0.88 ± 0.08	0.55 ± 0.21	0.92 ± 0.07	0.84 ± 0.10
Posterior Cingulate	0.78 ± 0.24	0.61 ± 0.2	0.87 ± 0.16	0.83 ± 0.13
Somatosensory Cortex	0.94 ± 0.06	0.74 ± 0.16	0.91 ± 0.08	0.87 ± 0.09
Thalamus	0.44 ± 0.39	0.55 ± 0.21	0.61 ± 0.21	0.68 ± 0.17

**Fig 3:**
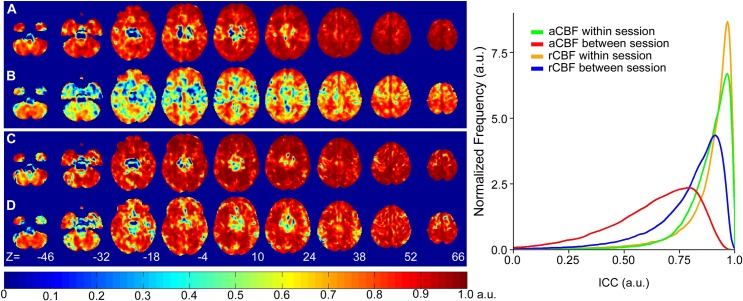
Voxel-wise whole brain intraclass correlation coefficient (ICC) maps calculated for absolute CBF (aCBF) and relative CBF (rCBF). (A) within-session aCBF, (B) between-session aCBF, (C) within-session rCBF, and (D) between-session rCBF. Histograms were generated from each ICC map.

Whole brain ICC maps and their corresponding histograms are displayed in Fig 3. The ICC maps depict excellent within-session grey matter reliability with values consistently above 0.75. This is also shown in the corresponding histograms: the distributions of ICC values for within-session reliability, shown in orange and green, are skewed towards a maximum value of 1.

Average voxel-wise within-session and between-session ICC values for all ROIs are given in Table 1B. Between-session ICC values for rCBF analyses were greater than the corresponding aCBF values. Within- and between-session rCBF ICC maps (Fig 3C and 3D) and region-averaged ICC values bore greater similarity compared to aCBF (Fig 3A and 3B).

### Arterial Transit Time Reproducibility

**Fig 4:**
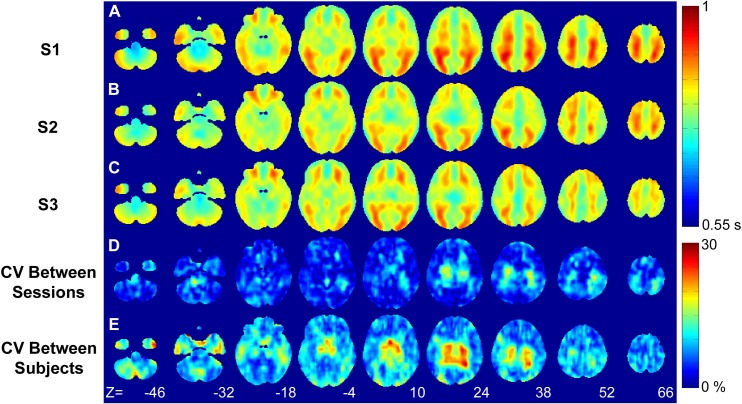
Whole brain group averaged (N = 7) arterial transit time maps for session 1 (A), session 2 (B), and session 3 (C). Voxel-wise between-session (D) and between-subject (E) coefficient of variance maps.

Group-averaged ATT maps for each of the three sessions are shown in Fig 4A–4C. Mean grey matter ATT values per session averaged across participants were 806 ± 45, 801 ± 35, and 796 ± 39 ms. There were no significant differences in mean grey matter ATT across sessions. Voxel-wise maps demonstrate regional heterogeneity with increased ATT in medial posterior and medial frontal regions.

The spatial patterns of the ATT maps were consistent across sessions, as demonstrated by the low between-session wsCV averaged over grey matter voxels (5.0 ± 2.7%). Additionally, there were no significant differences in voxel-wise ATT values across sessions. Variability between participants was higher, with a mean voxel-wise grey matter CV of 9.7 ± 3.5%. Voxel-wise between-subject maps (Fig 4) showed increased variability in the medial regions of the brain, while cortical grey matter remained more homogeneous

### Reproducibility of Longitudinal Motor Task Activation

**Fig 5:**
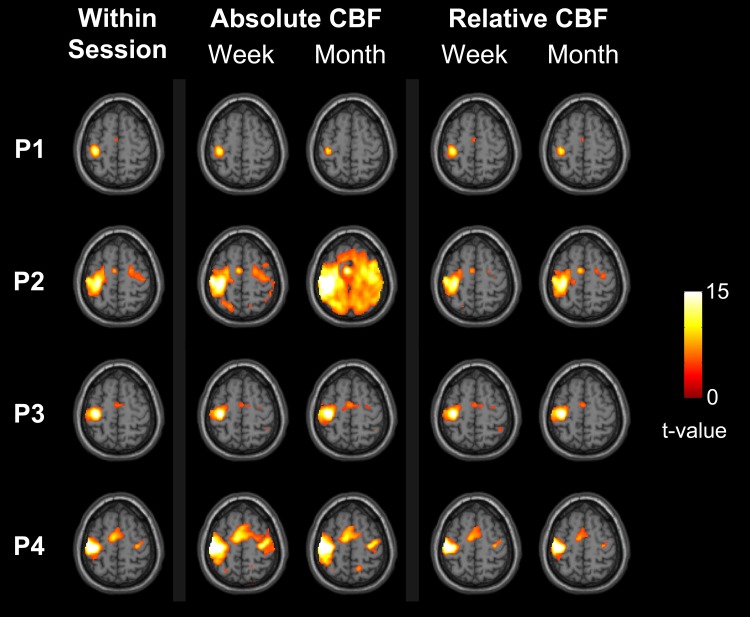
Representative sample of regional CBF changes associated with finger tapping overlaid on a T1-weighted MNI template brain. Activation maps were generated for: (a) within-session aCBF images, (b) rest and task aCBF images separated by a week and (c) aCBF images separated by a month, (d) rCBF images separated by a week and (e) rCBF images separated by a month. Regions in colour represent voxels that survived the statistical threshold after correction for family wise error (p < .05, FWE).

A representative sample of motor activation patterns from the aCBF and rCBF analyses is shown in Fig 5. From the within-session analysis, activation was detected in the primary motor cortex in all participants and also in the supplementary motor cortex in 6 participants and in the cerebellum in 3. In general, there was good agreement in the spatial pattern of activation generated using within- and between-session data, particularly after normalizing the perfusion images by grey matter CBF.

### Precision of Motor Activation

**Fig 6:**
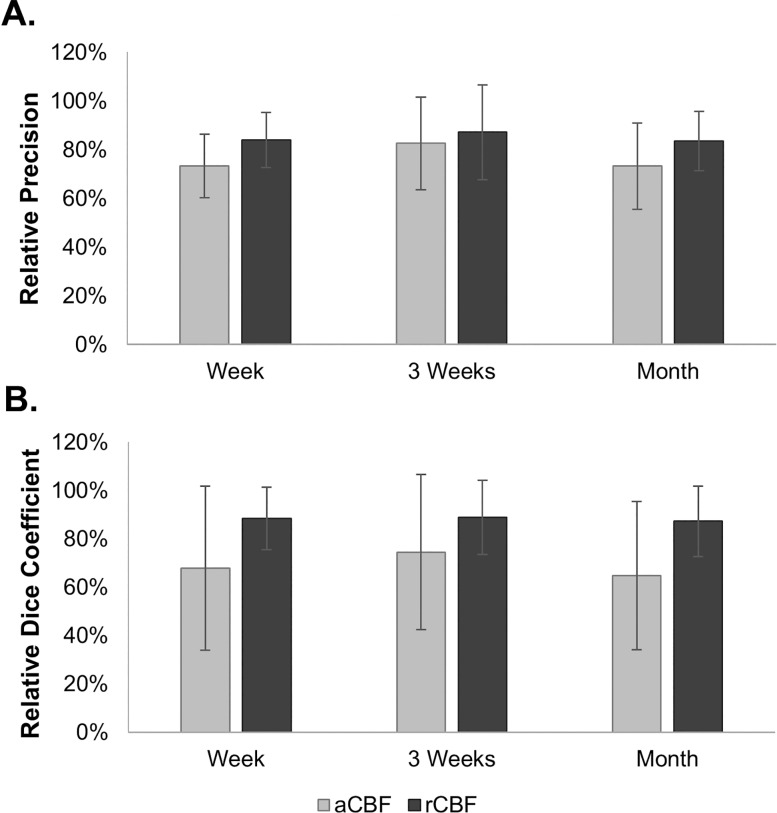
**Between-session (A) precision and (B) Dice coefficients expressed as a percent of within-session**_**DR**_ **value.** Absolute CBF is shown in light grey bars and relative CBF is shown in dark grey.

Mean within-session activation precision was 90 ± 7%. The precision was reduced to 70 ± 12%, 79 ± 13% and 70 ± 12% when the rest and task aCBF images were separated by a week, 3 weeks, and a month, respectively. The values using the rCBF images were 75 ± 15% (one-week), 78 ± 16% (3-week), and 75 ± 13% (one-month). Between-session precision relative to within-session precision for both aCBF and rCBF are shown in Fig 6. Overall, the precision of the rCBF activation maps was significantly greater than for aCBF. However, there was no significant difference in precision across sessions for either aCBF or rCBF.

### Dice Coefficient

The Dice coefficient based on the comparison of the within-session activation maps was 0.75 ± 0.07. The between-session values were 0.47 ± 0.19, 0.52 ± 0.11 and 0.45 ± 0.13 using aCBF activation maps separated by a week, 3 weeks and a month, respectively. Similarly, the values were 0.67 ± 0.13 (1 week), 0.67 ± 0.12 (3 weeks), and 0.66 ± 0.13 (1 month) from the rCBF activation maps. The between-session Dice coefficients relative to the within-session coefficients are shown in Fig 6. Dice coefficients measured with rCBF activation were significantly greater than aCBF, but there were no significant differences between sessions in either case.

## Discussion

The results of this study provide an assessment of the ability of ASL to detect voxel-wise changes in CBF across sessions separated by up to a month within an individual. First, we showed that between-session reproducibility and reliability were comparable to within-session values, particularly after removing the effects of day-to-day variations in global CBF. Second, ATT values were consistent across the three sessions and between-session variability was smaller than between-subject variability, indicating that ATT effects on monitoring longitudinal CBF changes were minimal. Finally, as a proof of concept, we demonstrated that activation maps similar to those generated within-session could be produced using rest and task CBF images from separate sessions.

Although post-processing registration helps reduce within-session alignment errors, the tolerance required for aligning images from different sessions is greater since registration errors will affect all images acquired in a session. This is in contrast to within-session motion that typically increases signal variance, rather than causing systemic artifacts. Accurate alignment of ASL images from different sessions is challenging because of the relatively large voxel size used to compensate for the low SNR. Consequently, slight alignment errors can lead to signal differences when comparing CBF images from separate sessions, and these errors can translate into false positive activation when applying standard statistical parametric mapping methods[5]. In this study, a relatively simple approach was used to mitigate registration errors. Personalized head molds were generated for each participant and were reused in subsequent imaging sessions to replicate the position of the head. The effectiveness of this approach is evident by the similarity in the magnitude of between-session and within-session transformation values. Although there was a greater tendency towards pitch rotations, all transformations required to align images from the separate sessions were less than 3 mm and 3°. The benefits of minimizing registration errors were evident by the good agreement in the variability measurements for within- and between-session analyses of relative CBF. Average voxel-wise wsCV in grey matter were 4.7 ± 4.5% and 5.7 ± 4.4% for the within- and between-session analysis, respectively. Likewise, the reliability, as indicated by the ICC measurements, was excellent for both within-session (0.89 ± 0.20) and between-session (0.84 ± 0.15) analyses. These results were reflected in the excellent agreement between the within- and between-session activation maps (Fig 5A versus Fig 5D & 5E).

As part of this study, efforts were made to also minimize day-to-day variations in CBF; however, comparing reliability and reproducibility measures of aCBF versus those for rCBF indicate that between-session analysis was affected by fluctuations in global CBF. Mean grey matter wsCV for aCBF was 75% greater than for rCBF, and the corresponding ICC decreased to 0.66 ± 0.19, a classification of good rather than excellent. These reductions likely reflected global CBF changes caused by diurnal fluctuations and state of arousal, which highlights the challenges of accounting for all sources of variability[2, 33–35]. As a caveat, the within-session wsCV was also improved by global normalization, which likely reflects changes in wakefulness and breathing pattern during an imaging session[36, 37].

The reproducibility and reliability maps (Figs 2 and 3) revealed spatial heterogeneity, particularly for the between-session analysis. The most noticeable feature was the higher variance in the centre of the head, corresponding to midbrain regions such as the thalamus (wsCV = 14.6 ± 7.3%). It has been suggested that increased variability in thalamic activity is a reflection of variability in arousal [31, 38]. Mezue et al. demonstrated that resting CBF in the thalamus decreased over 30 minutes, suggesting a decrease in attentional processing over time. To add, the thalamus is populated with large arteries which could have contributed pulsatile noise. However, in the current study, it is unlikely that thalamic activity is the sole contributor to the increased variability as the area extends beyond its borders. The most plausible cause in this study, however, is related to the 3D GRASE sequence. Single-shot 3D imaging was implemented to provide fast acquisition with good spatial coverage and SNR, which is advantageous for functional applications [15, 39]. However, it is susceptible to axial signal wrap-around and through-plane blurring [40, 41]. The greater between-session variance observed in the centre of the head caused by through-plane blurring could affect the results in applications interested in midbrain regions. For example in the study of chronic pain, the thalamus plays a key role in the modulation of nociceptive information in the acute and chronic phase[42, 43]. One possible solution would be to use a multi-shot 3D GRASE sequence to improve the phase encoding along the axial direction and reduce the acquisition window [41, 44].

Recent studies have identified spatial heterogeneity in ATTs as a confounder for measuring CBF accurately [5, 45]. Although multi-PLD sequences have been used to image ATT and CBF simultaneously, the trade-off is suboptimal SNR for perfusion imaging and increased acquisition times [46]. In the current study, a low-resolution ATT sequence was implemented because ATT values are fairly homogeneous within large vascular territories [11, 45]. The similarity in the appearance of the group-wise ATT maps generated per session and the low between-session wsCV values (Fig 4) demonstrated that regional ATT values were consistent across sessions. Furthermore, no significant voxel-wise ATT changes between sessions were found, and there was good agreement in the average grey matter ATT values from the three sessions (806 ± 45, 801 ± 35 and 796 ± 39 ms, respectively). These results show that fluctuations in ATTs are not a confounder in longitudinal CBF studies, at least in healthy individuals, provided the appropriate PLD is chosen. Clearly, monitoring ATT in studies involving older participants or patients with vascular disease would be prudent, particularly considering that low-resolution ATT images can be acquired in only a few minutes[46]. Note, these values are smaller than previously reported [20, 46] because the GRASE sequence did not include vascular crusher gradients to suppress signal contributions from feeding arteries. Consequently, these ATT values represent the delay from the labeling plane to the imaging voxels and not to the capillary bed.

The similarity in the within- and between-session measures of reproducibility and reliability indicate that ASL should have sufficient statistical power to detect longitudinal changes in regional CBF. To demonstrate this, statistical parametric mapping was performed on rest and motor activation ASL data sets from sessions separated by up to a month. This approach represents a proof of concept of the ability of ASL to detect inter-sessional activation. Since the same task data were used for the within- and between-session analyses, the resulting activation maps should ideally be the same, provided additional between-session sources of variance were minimal. Visual inspection of the activation maps generated before (aCBF) and after global normalization (rCBF) indicates that fluctuations in global CBF can reduce the ability to detect the true activation[2]. This was evident in participant 2, in which the activation detected at 1 month included most of the brain due to a 12.4 ml/100g/min increase (25.5%) in global CBF between the two sessions (Fig 5). Activation in the primary motor cortex and supplementary motor region could be identified by larger t-scores in these regions compared to the rest of the brain. Normalizing the CBF images from each session by their global value substantially reduced the number of false positives, and the resulting between-session activation map appeared very similar to the original within-session map.

Despite the similarities in the appearance of the within and between-session rCBF activation maps shown in Fig 5, displaying a single slice does not properly assess the extent of false activation. Instead, the quality of between-session activation was characterized by first measuring the precision. This was determined from the number of activated voxels in motor-related regions as defined by the TP mask created using the within-session activation (i.e., true activation) and those in the rest of the brain (i.e., false activation). In agreement with the noise metrics, on average, there was a 15% decrease in precision between-sessions. In other words, even with a month separation between rest and task images, there was less than a 16% increase in the number of false positives. Although this measure provides a means of assessing the magnitude of false activation, using the task data to define the TP region could have introduce a bias. To assess this possibility, precision was also calculated using anatomically defined sensorimotor regions as the TP ROI. Good agreement was obtained, with a 15% decrease in precision when comparing task and rest data sets separated by a month (Table B in S1 File).

In addition to precision, the Dice coefficient was used to assess the similarity between within- and between-session activation maps. Since true activation is difficult to define, the Dice coefficient, using the two activation sets from the same session, was used as a reference (0.75 ± 0.11). A 12% decrease in common voxels was found from the between-session analysis, but there were no significant differences across the sessions. Considering that the fraction of true activated voxels was approximately 1% of the total number of grey matter voxels, these precision and Dice coefficient estimates highlight the ability of ASL to detect longitudinal changes in CBF, particularly if the confounding effects of variations in global CBF are removed. To assess if these results would be affected by using a less stringent statistical threshold, activation maps were also generated based on the False Discovery Rate (*p*>0.05) instead of FWE. The resulting relative precision and Dice coefficients were within 9% of the values reported in this study (Tables B and C in S1 File).

The minimal detectable CBF change in a given voxel was estimated based on the paired sample *t*-test equation (i.e. ΔCBF_min_ = (SDΔ_CBF_ /√n)/*t*_crit_), where the critical *t*-statistic (*t*_crit_) was estimated using the FWE-corrected *t*-threshold generated by the voxel-wise analysis, SDΔ_CBF_ was calculated from the MATLAB implemented ANOVA, and n was the number of perfusion images per run. For the within-session analysis, this threshold was approximately 3%, while for the between-session analysis it was 7% for the aCBF images and 4% for the rCBF images. These thresholds are considerably smaller than the high percent signal increase (~40–60%) reported for motor task activation[47, 48], but are in-line with previous calculations[49]. The magnitude of the between-session thresholds indicates that ASL should be capable of detecting longitudinal changes in brain function, such as those caused by pain[50], which are associated with smaller CBF changes than those produced by a motor sensory task. This is in line with a recent study showing significant correlation between regional CBF changes in the thalamus, amygdala and primary somatosensory cortex and changes in pain perception monitored over a 7–21 day period[1].

## Limitations

While the results of this study showed promise, there are some considerations for future clinical applications. First, the participants were young and healthy adults, and caution must be exercised in extrapolating these results to other demographics. In particular the inter-sessional stability of the ATT measurements may not be true for older individuals and patients with cerebrovascular disease[40]. Second, if global CBF is not intact, normalizing to global grey matter CBF can introduce biases in activation maps[33]. One potential solution would be to normalize the CBF images by a region considered unaffected by the condition of interest. However, in instances where areas unaffected by the disease are not known a priori, explicit use of aCBF may be necessary[36]. Third, the steps employed in this study to reproduce the head position across sessions were effective but time consuming, adding about 20 minutes to make an individual head mold in the first session. Recent studies have demonstrated the potential of online automatic planning software[9] to replicate imaging position between sessions. However, the accuracy of such software relative to that achieved in the current study using head molds needs to be confirmed. Finally, it would be useful to increase the time between rest and task sessions given that CBF monitoring over periods greater than a month would be more relevant to studying disease progression.

## Conclusion

This study demonstrated that ASL has the sensitivity to detect motor activation over periods extending up to a month on an individual basis. At the voxel-wise level, we demonstrated low variability in resting CBF and similar within- and between-session activation maps after removing variations in basal blood flow. Furthermore, ATT was not a confounder to the reproducibility of CBF. These results demonstrate the feasibility of conducting voxel-wise analysis of CBF images acquired on different days and highlights the potential of ASL for longitudinal studies to assess changes in brain function related to disease processes and treatment.

## Supporting Information

S1 File **Fig A.** Pictorial representation of: (A) data acquisition and (B) data analysis. Data were acquired in 3 sessions, where blue red and orange represent sessions 1 through 3 respectively. Each session consisted of two runs, where each run was comprised of a ~5 minute resting period (indicated by darker shade) and a ~5 minute sequential finger tapping task period (indicated by the lighter shaded colour). Contrasts were generated by concatenating task data with rest:
Within-session
ex. Session 1 Run 1 Rest vs. Session 1 Run 1 Taskwithin-session different run (within-session_DR_)
ex. Session 1 **Run 2** Rest vs. Session 1 **Run 1** Taskbetween-sessions
ex. 1 week: **Session 2** Run 1 Rest vs. **Session 1** Run 1 Taskex. 1 month: **Session 3** Run 1 rest vs. **Session 1** Run 1 Task)
For precision and dice analysis, activation data generated using the same task data were compared to each other (i.e down each column). That is to say, the task data remained the same while the rest data was within-session, within-session_DR_ or between-session. The precision and dice coefficients were then averaged together based on the separation between rest and task. A similar analysis was performed for run 2 data. **Table A.** Background suppression timing used for ASL and ATT mapping sequences. **Table B.** Precision measured using the activated region defined by the family wise error rate (FWE), false discovery rate (FDR), or an anatomically defined motor region (AAL) as the true positive region. Between-session precision is expressed relative to within-session_DR_ precision. **Table C.** Between-session Dice coefficients relative to within-session_DR_ measured with FWE and FDR statistical thresholds.
(PDF)
