# Supplementary material for: Mapping Long-Term Functional Changes in Cerebral Blood Flow by Arterial Spin Labeling
Source: PLoS One. 2016 Oct 5;11(10):e0164112. doi: 10.1371/journal.pone.0164112 (PMC5051683; doi:10.1371/journal.pone.0164112)
Supplement: S1 File — Fig A. Pictorial representation of: (A) data acquisition and (B) data analysis. Data were acquired in 3 sessions, where blue red and orange represent sessions 1 through 3 respectively. Each session consisted of two runs, where each run was comprised of a ~5 minute resting period (indicated by darker shade) and a ~5 minute sequential finger tapping task period (indicated by the lighter shaded colour). Contrasts were generated by concatenating task data with rest: Within-session ex. Session 1 Run 1 Rest vs. Session 1 Run 1 Taskwithin-session different run (within-sessionDR) ex. Session 1 Run 2 Rest vs. Session 1 Run 1 Taskbetween-sessions ex. 1 week: Session 2 Run 1 Rest vs. Session 1 Run 1 Taskex. 1 month: Session 3 Run 1 rest vs. Session 1 Run 1 Task) For precision and dice analysis, activation data generated using the same task data were compared to each other (i.e down each column). That is to say, the task data remained the same while the rest data was within-session, within-sessionDR or between-session. The precision and dice coefficients were then averaged together based on the separation between rest and task. A similar analysis was performed for run 2 data. Table A. Background suppression timing used for ASL and ATT mapping sequences. Table B. Precision measured using the activated region defined by the family wise error rate (FWE), false discovery rate (FDR), or an anatomically defined motor region (AAL) as the true positive region. Between-session precision is expressed relative to within-sessionDR precision. Table C. Between-session Dice coefficients relative to within-sessionDR measured with FWE and FDR statistical thresholds. (PDF) [file pone.0164112.s001.pdf]

Supporting Information

Mapping Long-Term Functional Changes in Cerebral Blood Flow by Arterial Spin Labeling

Tracy Ssali[1,2*], Udunna C Anazodo[1, 2], Yves Bureau[1], Bradley J MacIntosh[3], Matthias Günther[4,5], Keith St Lawrence[1, 2]

[1]Lawson Health Research Institute, London, ON, Canada.
[2]Department of Medical Biophysics, Western University, London, ON, Canada.
[3] Sunnybrook Health Sciences Centre, Toronto, ON, Canada.
[4] Fraunhofer Institute for Medical Image Computing MEVIS, Bremen, Germany
[5] Mediri GmbH, Heidelberg, Germany

*** Corresponding author:** Tracy Ssali, Electronic mail: tssali@uwo.ca.

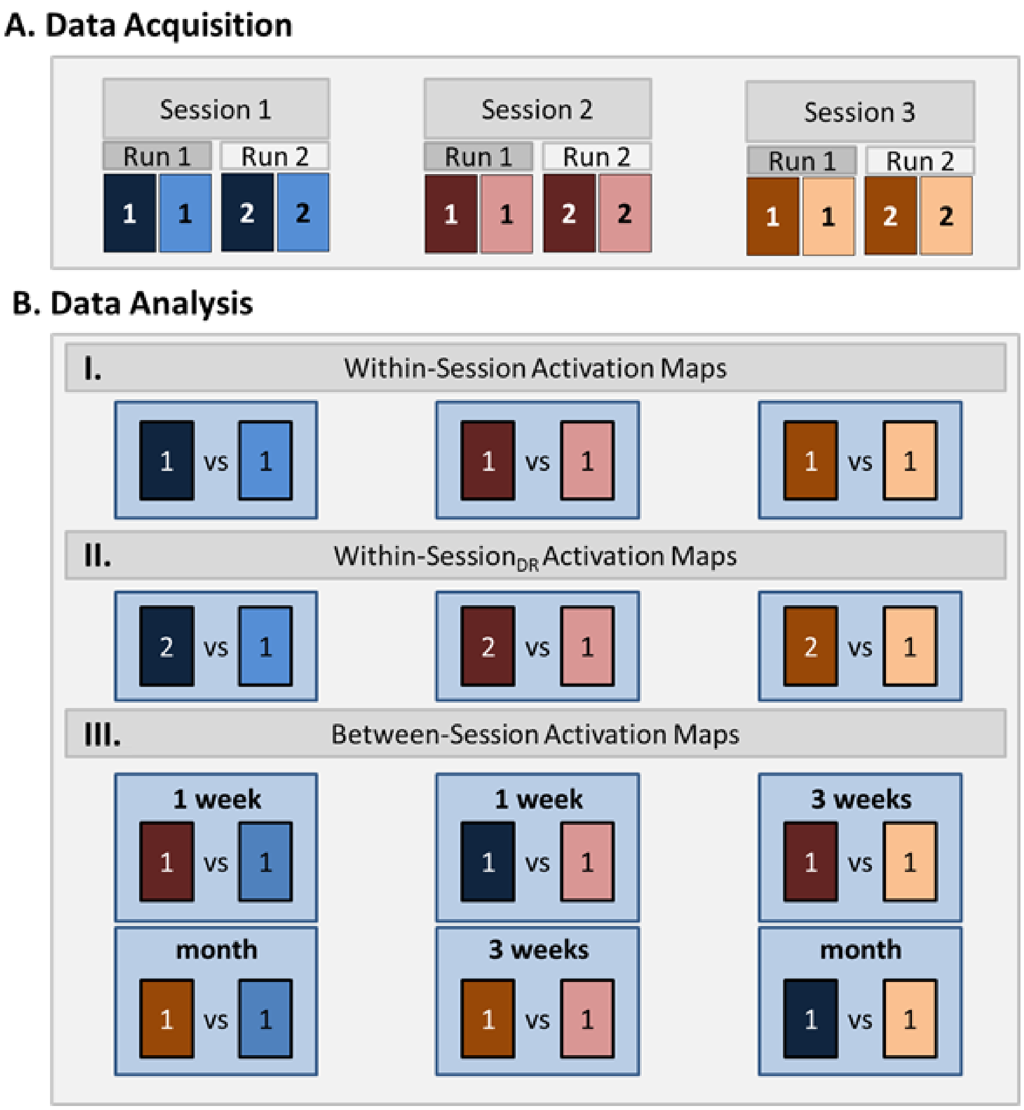

Fig A. Pictorial representation of: (A) data acquisition and (B) data analysis. Data were acquired in 3 sessions, where blue red and orange represent sessions 1 through 3 respectively. Each session consisted of two runs, where each run was comprised of a ~5 minute resting period (indicated by darker shade) and a ~5 minute sequential finger tapping task period (indicated by the lighter shaded colour).

Contrasts were generated by concatenating task data with rest:

(I.) Within-session

  a. ex. Session 1 Run 1 Rest vs. Session 1 Run 1 Task

(II.) within-session different run (within-session$_{DR}$)

  a. ex. Session 1 **Run 2** Rest vs. Session 1 **Run 1** Task

(III.) between-sessions

  a. ex. 1 week: **Session 2** Run 1 Rest vs. **Session 1** Run 1 Task
  b. ex. 1 month: **Session 3** Run 1 rest vs. **Session 1** Run 1 Task)

For precision and dice analysis, activation data generated using the same task data were compared to each other (i.e down each column). That is to say, the task data remained the same while the rest data was within-session, within-session$_{DR}$ or between-session. The precision and dice coefficients were then averaged together based on the separation between rest and task. A similar analysis was performed for run 2 data.

Table A. Background suppression timing used for ASL and ATT mapping sequences.

Post Labeling Delay	Inversion Pulse 1 (ms)	Inversion Pulse 2 (ms)
ASL		
1200	372	956
ATT		
700	198	543
1300	410	1041
1900	666	1563
2500	965	2102
3100	1310	2657

Table B. Precision measured using the activated region defined by the family wise error rate (FWE), false discovery rate (FDR), or an anatomically defined motor region (AAL) as the true positive region. Between-session precision is expressed relative to within-session$_{DR}$ precision.

	aCBF			rCBF		
	**FWE**	**FDR**	**AAL**	**FWE**	**FDR**	**AAL**
**Week**	74 ± 13%	69 ± 13%	78 ± 14%	84 ± 11%	76 ± 11%	87 ± 9%
**3 Weeks**	84 ± 19%	84 ± 27%	87 ± 12%	88 ± 20%	84 ± 19%	91 ± 19%
**Month**	75 ± 17%	73 ± 27%	80 ± 11%	84 ± 17%	82 ± 12%	85 ± 12%

Table C. Between-session Dice coefficients relative to within-session$_{DR}$ measured with FWE and FDR statistical thresholds.

	**aCBF**		**rCBF**	
	**FWE**	**FDR**	**FWE**	**FDR**
**Week**	74 ± 33%	71 ± 48%	88 ± 12%	83 ± 13%
**3 Weeks**	79 ± 32%	82 ± 54%	90 ± 18%	87 ± 15%
**Month**	69 ± 30%	69 ± 50%	87 ± 16%	87 ± 15%
